# StaBle-MambaNet: structure-aware and blur-guided lane detection with Mamba

**DOI:** 10.3389/frai.2025.1687983

**Published:** 2025-11-20

**Authors:** Xiaoyu Zhang, Hongwei Huang, Xiting Peng, Xiaoling Zhang, Lexi Xu, Yang Yang

**Affiliations:** 1 School of Artificial Intelligence, Shenyang University of Technology, Shenyang, China; 2 Shenyang Key Laboratory of Industrial Intelligent Chip and Network System Innovation Application, Shenyang, China; 3 School of Information Science and Engineering, Shenyang University of Technology, Shenyang, China; 4 Shenyang Key Laboratory of Advanced Computing and Application Innovation, Shenyang, China; 5 Research Institute, China Unicom China United Network Communications Corporation, Beijing, China

**Keywords:** lane detection, blurred scenes, structural confirmation, feature completion, temporal modeling, blur-aware representation

## Abstract

The perception system constitutes a critical component of autonomous driving, due to factors such as high-speed motion and complex illumination, camera-captured images often exhibit local blurring, leading to the degradation of lane structure clarity and even temporary disappearance of lane markings, which severely compromises the accuracy and robustness of lane detection. Traditional approaches typically adopt a two-stage strategy of “image enhancement followed by structural recognition” Initially, the entire image undergoes deblurring or super-resolution reconstruction, followed by lane detection. However, such methods rely on the quality of full-image restoration, exhibit low processing efficiency, and struggle to determine whether the disappearance of lane markings is genuinely caused by image blurring. To address these challenges, this paper proposes an Inter-frame Stability-Aware Blur-enhanced Mamba Network (StaBle-MambaNet), which identifies blurred regions and assesses the presence of potential lane structures without relying on full-image restoration. The method first localizes blurred areas and employs a Structure-Aware Restoration Module to perform directional extrapolation and completion for potential lane line regions. Subsequently, the Blur-Guided Consistency Reasoning Module evaluates structural stability to identify genuine lane regions. Finally, enhanced features are constructed into a spatially continuous token sequence, which is fed into a lightweight state-space model, Mamba, to model the dynamic feature variations in blurred regions while preserving the vertical structural evolution of the image. Experimental results demonstrate that StaBle-MambaNet significantly outperforms existing mainstream methods across multiple public lane datasets (e.g., CULane and CurveLanes), particularly under challenging conditions such as nighttime, occlusion, and curved lanes, exhibiting clear advantages in both detection accuracy and structural stability.

## 1 Introduction

With the rapid development of intelligent driving technology, lane detection, as one of the core perception tasks, holds significant importance for achieving path planning and safety control (Luo et al., 2025; Yurtsever et al., 2020). Deep learning techniques have substantially improved the accuracy of lane detection by leveraging the powerful feature extraction capabilities of Convolutional

**Figure 1:**
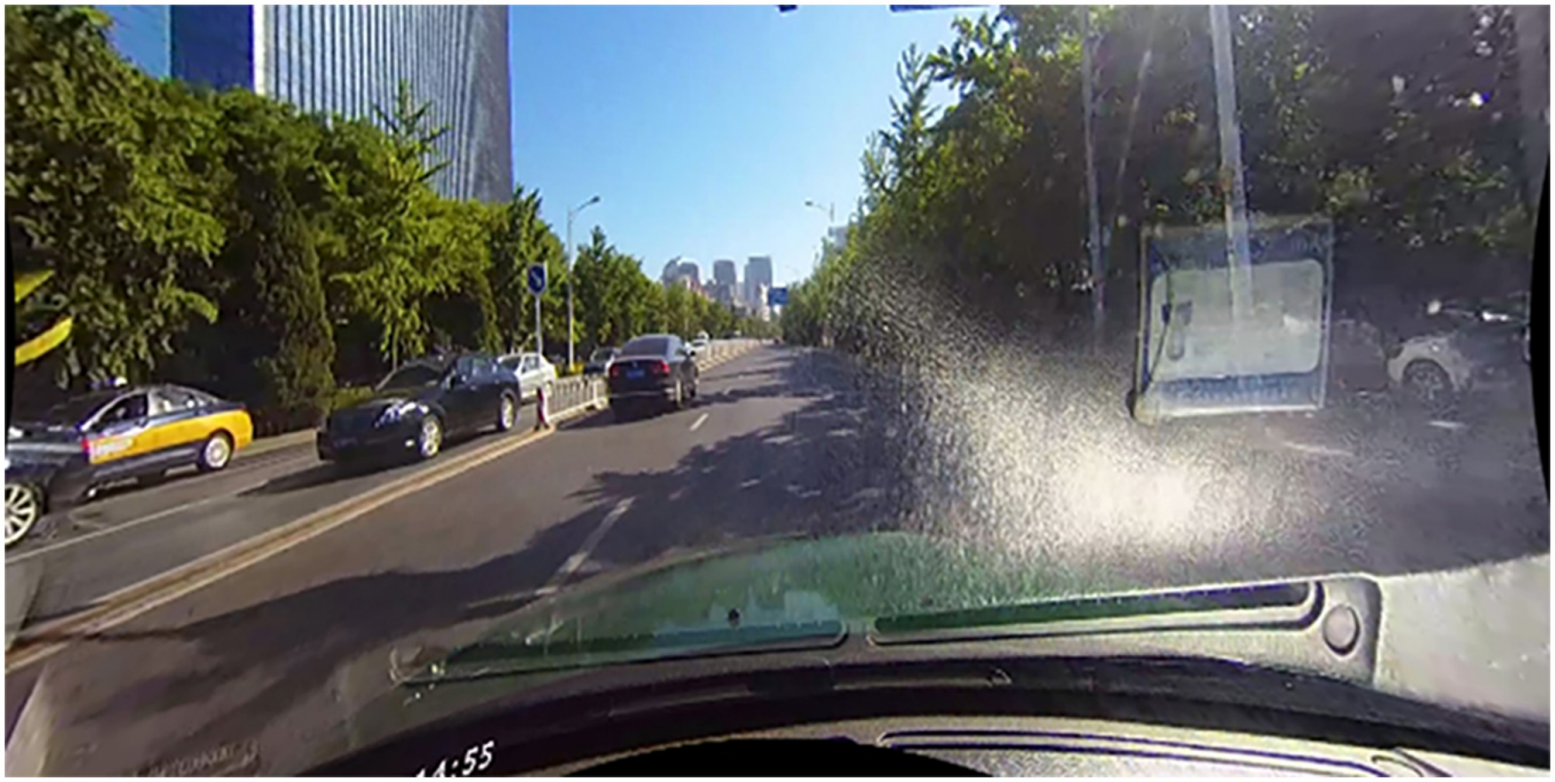
Lane blurring from overexposure and motion obscures markings, reducing detection accuracy in challenging conditions (reproduced with permission from the CULane dataset, [https://xingangpan.github.io/projects/CULane.html]), licensed under CC-BY-NC.

Neural Networks (CNNs) to extract road regions from static images or video frames, enabling structured modeling through approaches such as semantic segmentation and keypoint fitting. Particularly under ideal conditions with clear structures and balanced illumination, various image-based lane detection methods have demonstrated strong recognition capabilities. However, during high-speed operation, cameras subjected to vibration, varying lighting conditions, or focal length drift often capture images containing blurred areas, motion ghosting, or even missing lane markings. Furthermore, challenges like adverse weather, backlighting environments impede continuous perception of lane structures. As shown in Figure 1 under high-speed or overexposed scenarios, lane structures may become invisible due to blur effects. These low-quality regions typically occur near actual lane boundaries, making it difficult for networks to determine structural authenticity and severely compromising detection continuity and reliability.

To mitigate interference from image blurring on lane perception, existing studies employ image enhancement or video modeling strategies to compensate for structural recognition impairment. One approach adopts an image restoration paradigm, utilizing deblurring networks, contrast enhancement, or super-resolution reconstruction to improve input quality, aiming to recover structural information within blurred areas via global image sharpening (Ji et al., 2022; Wang et al., 2023a; Dong and Lan, 2024). Yet such methods suffer from lengthy processing pipelines after whole-image restoration, failing to focus specifically on lane regions while exhibiting performance degradation under restoration failure or excessive noise interference (Mercy et al., 2022). Alternative methods incorporate temporal modeling by leveraging inter-frame information for feature completion or sequence modeling using architectures like ConvLSTM and Transformer to enhance temporal coherence and global context understanding. Though partially addressing local structure gaps, these approaches indiscriminately model all regions without considering blur specificity, leading to resource waste, model bias, redundant information interference, attention dilution, and temporal drift issues (Yang et al., 2022). In recent years, the Mamba model has emerged as a structurally efficient State Space Model architecture. By employing dynamic weight control and a sliding window mechanism, it can maintain global modeling capabilities while significantly reducing computational complexity and enhancing the modeling effect of long-term dependencies.

This paper proposes an Inter-frame Stability-Aware Blur-enhanced Network (StaBle-MambaNet) targeting blurred regions. The method proposed in this paper differs from these paradigms in two main aspects. First, by focusing only on ambiguous regions, it avoids the computational overhead and potential artifacts associated with global restoration. Second, unlike other approaches that may indiscriminately attempt to enhance any degraded region, this method introduces a crucial validation step: before any completion or modeling, it first determines whether the ambiguous region contains genuine lane structures with temporal consistency. This ensures that network resources are utilized only for reliable structural information. A Blur-Guided Consistency Reasoning Module evaluates region importance, while a Structure-Aware Restoration Module performs directional extrapolation for lane completion. Finally, a Blur-enhanced Temporal Modeling Mamba (BTM-Mamba) selectively enhances and models only trustworthy blurred regions. Unlike holistic image restoration pipelines and generic sequence encoders (e.g. Transformer variants), this study implements blurred region localization, lane structure stability testing, completion restricted to validated areas, and applies state-space temporal modeling specifically to corresponding labels. This design circumvents reliance on global reconstruction while mitigating indiscriminate temporal aggregation across time dimensions. The core architecture centers on structural inference of ambiguous features coupled with selective temporal modeling, rather than executing global enhancement or uniform sequence processing across entire frames. This approach minimizes redundant interference while improving modeling efficiency and stability for latent structures in degraded scenes, effectively addressing limitations in structural discrimination, focused modeling, and computational adaptability.

The main contributions are summarized as follows:

- StaBle-MambaNet: integrate blur awareness, structural judgment, and adaptive pathways into a unified pipeline for differentiating degradation artifacts from true road structure variations under motion blur.
- Dual-module stability mechanism: the Structure-Aware Restoration Module (SARM) and Blur-Guided Consistency Reasoning Module (BCRM) jointly compute inter-frame feature discrepancies to assess the reliability of ambiguous regions and generate stability masks for selective completion.
- Temporal modeling: blur-enhanced temporal modeling module (BTMM) with Mamba captures long dependencies through a state-space formulation applied to stability-weighted features, providing efficient temporal representation without indiscriminate sequence aggregation, while multi-objective optimization (SSML) combines L_*cons*_ and slope-sensitive LaneIoU for accuracy.
- Experiments across public datasets validate our method's robustness and continuity in identifying lane structures under blurred conditions, demonstrating superior generalization and task adaptability.

The remainder of this paper is organized as follows. Section 2 reviews related work on lane detection, temporal modeling, and structural completion under degraded visual conditions. Section 3 presents the proposed StaBle-MambaNet framework in detail, including the Structure-Aware Restoration Module, Blur-Guided Consistency Reasoning Module, and Blur-enhanced Temporal Modeling Mamba. Section 4 describes the experimental setup, datasets, evaluation metrics, and performance comparison with existing methods, followed by comprehensive ablation studies. Finally, Section 5 concludes the paper and outlines directions for future work.

## 2 Related work

### 2.1 Lane detection

In 2D lane detection based on camera images, deep learning-based methods can be categorized into five types according to their detection heads: classification-based approaches determine whether features belong to lanes through classification. For instance, DVCNN partitions the input image and classifies blocks as either lane or non-lane; SLTNet performs block-wise classification on bird's-eye-view (BEV) images for lane detection (Song et al., 2023; Xie, 2023). Anchor-based methods generate predefined anchor lines and regress their offsets relative to ground-truth lane annotations. LineCNN creates anchor lines with varying slopes from boundary pixels of the feature map; CurveLane-NAS generates small anchor lines on multi-scale feature maps and clusters them, making it suitable for curved lanes (Qin et al., 2022; Gao and Lu, 2025). Row-wise-based methods select representative pixels per row and cluster them to form lanes. UFLD flattens the feature map and employs a multilayer perceptron (MLP) to select row-wise pixels; CondLaneNet enhances accuracy through instance segmentation and dynamic kernel parameter regression (Liu Y. et al., 2021; Chai et al., 2024). Polynomial-based methods directly predict polynomial coefficients to represent lanes. PolyLaneNet uses an MLP to predict second-order polynomial coefficients; PRNet adopts piecewise polynomials to detect complex curved lanes (Su et al., 2021). Segmentation-based methods utilize semantic segmentation to identify lane pixels and group them into complete lanes. SCNN employs spatial CNNs to aggregate features from neighboring pixels; VPGNet integrates lane detection with vanishing point estimation in a multi-task learning framework, with some approaches further refining results via RANSAC or polynomial fitting (Kaushal et al., 2023; Gao et al., 2023). The method proposed in this paper falls within the segmentation-based category, introducing mechanisms for ambiguous region identification, structural verification, and inter-frame feature modeling upon conventional semantic segmentation. Unlike existing segmentation methods that rely solely on pixel-wise mask outputs, our approach incorporates structural extrapolation, heatmap differential analysis, and state-space modeling (Mamba) within a structural-aware pathway, enabling the network to determine whether ambiguous regions correspond to actual lane markings and subsequently perform structural completion and dynamic modeling.

### 2.2 Temporal modeling with state space models

State Space Models (SSMs) have long served as an efficient tool for temporal modeling, widely employed to capture long-range dependencies in dynamic systems. However, conventional SSMs typically rely on recursive inference, resulting in low computational efficiency and difficulties in handling complex tasks involving long sequences and high-dimensional inputs (Gu and Dao, 2023). To address this limitation, the Structured State Space Sequence Model (S4) significantly improved modeling efficiency by introducing convolution over latent states, thereby validating for the first time the effectiveness of structured state space models in long-sequence modeling (Somvanshi and Islam, 2025). Building upon this foundation, the Mamba model was proposed as an enhanced variant of S4. By incorporating a dynamic weighting mechanism and a hardware-friendly parallel scan architecture with sliding windows, Mamba achieves linear time complexity in sequence modeling (Gu and Dao, 2023; Liu et al., 2024). The Mamba block consists of a gated MLP, a state-space transformation (an improved S4), and residual connections, employing the SiLU or Swish activation function to effectively integrate sequential and spatial modeling capabilities (Bansal et al., 2024). Specifically, Mamba designs the original state matrix A, input matrix B, output matrix C, and step size S as functions of the input sequence, dynamically generating parameters through projection mechanisms that adapt to the input data, thereby enhancing the model's adaptability to sequential variations (Rando et al., 2025; Liang et al., 2024). Equation 16 enables Mamba to model temporal dependencies in blurred lane regions by dynamically adjusting matrices A, B, C, and step size S, enhancing detection robustness under varying conditions.


$$h^{'}\left(t\right)=Ah\left(t\right)+Bx\left(t\right)$$ (1)

$$\begin{array}{l} S,\text{A},\text{B},\text{C}=\text{Linear}\left(x\left(t\right)\right) \end{array}$$ (2)

Mamba exhibits exceptional performance in sequence modeling tasks. In this work, we incorporate Mamba as the core module for inter-frame structural modeling within lane detection systems, aiming to extract spatiotemporal dynamic features across consecutive frames. This approach enhances structural restoration and temporal awareness capabilities, particularly benefiting robust judgment of structural stability and information completion processes under degraded visibility conditions (He and Ji, 2025; Lin and Chiang, 2024; Zhang et al., 2025).

### 2.3 Structure completion and contextual inference

Structure completion constitutes a critical research direction in lane detection tasks, aiming to generate plausible predictions and restorations for visually incomplete or obscured lane structures. Early approaches predominantly relied on conventional image processing techniques–such as line fitting after edge detection or RANSAC-based polynomial approximation–to extrapolate trajectories of lane markings; however, these methods exhibited limited robustness when confronted with blurry occlusions or complex curvilinear configurations (Talib et al., 2013). With the advancement of deep learning, studies have explored temporal modeling mechanisms for dynamic structural completion of lane features. At finer granularities of structural recovery, incorporating edge priors has demonstrated efficacy in enhancing completion accuracy (Zakaria et al., 2020). Techniques from image inpainting domains, exemplified by EdgeConnect (Nazeri et al., 2019), which involve extracting edge maps followed by contextual synthesis, have markedly improved reconstruction fidelity. This paradigm holds significant relevance for lane detection: as high-frequency structural cues, lane edges provide stable orientational guidance that informs missing region interpolation. Furthermore, leveraging semantic context has emerged as an evolving trend–certain methodologies integrate driveable area constraints, vanishing point cues, or road topology priors to enable principled inference of fragmented lane instances (Xiao et al., 2021; Xi et al., 2024). This paper addresses limitations in existing methods by focusing on localized blur assessment and temporal consistency, enhancing lane detection in degraded conditions. It effectively discerns whether ambiguous regions contain stable lane configurations, thereby facilitating adaptive structural completion and optimized modeling while enhancing perceptual robustness and recovery performance under degraded visibility conditions.

### 2.4 Lane detection in blurred scenarios

Handling visual degradation caused by blur is a critical challenge in lane detection. Existing approaches can be broadly categorized into two main paradigms: two-stage restoration-based methods and end-to-end blur-robust models.

The first paradigm follows a “restore-then-detect” pipeline. These methods treat blur as a pre-processing problem, applying a global image enhancement algorithm to the entire input frame before feeding it to a downstream lane detector. Techniques range from classic contrast enhancement to more advanced deep learning-based deblurring networks and generative adversarial networks (Liu et al., 2022). While straightforward, this approach has notable drawbacks. Global restoration is computationally intensive, often compromising the real-time requirements of autonomous driving. Furthermore, these task-agnostic enhancement methods can introduce unexpected artifacts or fail to recover the specific high-frequency details crucial for lane markings, making the final detection accuracy heavily reliant on the quality of the initial restoration step.

The second paradigm focuses on building end-to-end models that are inherently robust to blur. This is often achieved by training networks on datasets augmented with various blur effects, encouraging the model to learn invariant features. Other approaches leverage temporal information from video sequences, using architectures like ConvLSTM (Lin et al., 2020) or Transformers to infer lane structures from adjacent, clearer frames (Wang et al., 2023b). While more efficient than two-stage methods, these end-to-end models often lack a targeted mechanism for ambiguous regions. They may attempt to model all areas indiscriminately, leading to wasted computation and potential attention dilution. Crucially, they typically do not possess an explicit component to verify whether a blurred region contains a genuine lane structure or is merely noise. This lack of a structural verification step can lead to unreliable completions, especially when scene changes occur.

## 3 Method

**Figure 2:**
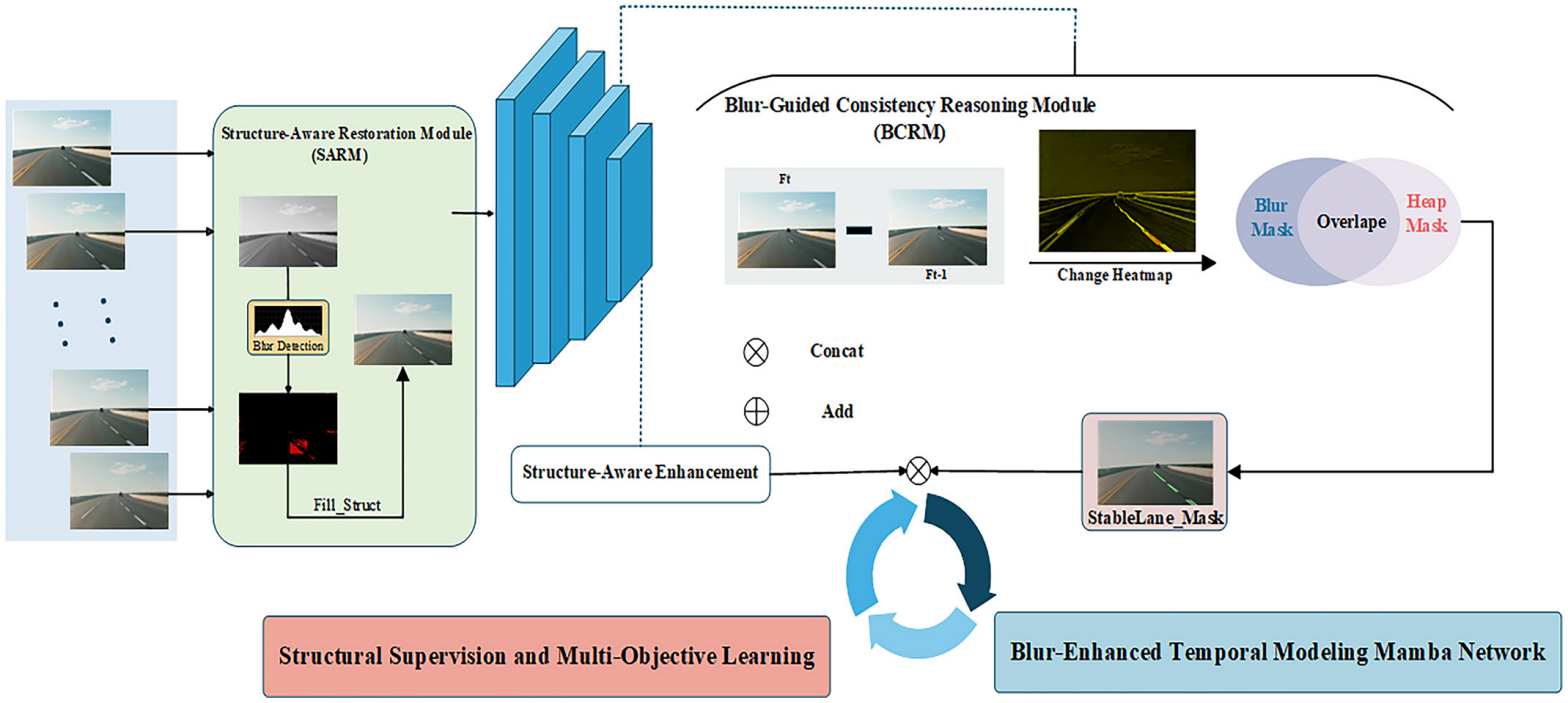
Conceptual framework. Structure-Aware Restoration Module (SARM), Blur-Guided Consistency Reasoning Module (BCRM), and Blur-Enhanced Temporal Modeling Mamba (BTM-Mamba).

The proposed framework is designed to intelligently handle blurred regions by first verifying structural integrity before committing to restoration and temporal modeling, a key distinction from conventional “restore-then-detect” pipelines. This paper proposes an Inter-frame Stability-Aware Blur-enhanced Mamba Network (StaBle-MambaNet) framework for lane detection, aiming to address the issue of information loss in blurred scenes. The overall architecture is illustrated in the figure and primarily consists of the following modules: (1) Structure-Aware Restoration Module (SARM), which performs blur region detection/restoration and structural completion within these areas; (2) Blur-Guided Consistency Reasoning Module (BCRM), leveraging inter-frame structural discrepancies to infer potential lane regions; (3) Blur-enhanced Temporal Modeling Mamba (BTM-Mamba), implementing cross-frame dynamic modeling of stable structures based on the Mamba architecture; (4) Structural Supervision and Multi-Objective Learning (SSML), employing composite loss functions for joint optimization of lane prediction outcomes. The overall pipeline is illustrated in Figure 2.

### 3.1 Structure-aware restoration module

First, perform blur analysis on the current input frame L_*t*_. Since regions with sharp details exhibit high variance in Laplacian responses while blurred areas demonstrate smooth, low-frequency variations, the Laplacian Variance method–based on the Laplacian operator–is employed to detect potentially blurred regions within the image (Bansal et al., 2016).

To detect regions suffering from motion blur, we compute the Laplacian variance over the input frame F_*t*_. A lower variance indicates a smoother region, likely due to blur. The blur score for each pixel is defined as follows:


$$\begin{array}{l} Var_{t}=\text{Var}\left(\text{Lap}\left(F_{t}\right)=\nabla^{2}F_{t}\right) \end{array}$$ (3)

$${\text{M}}_{blur}(i,j)=\{\begin{array}{ll} 1 & {\text{if Var}}_{t}(i,j)<\tau \\ 0 & \text{otherwise} \end{array}$$ (4)

where (*i, j*) denotes the pixel coordinates, and τ represents the blur determination threshold. For ambiguous regions, this paper employs a directional vector linear extrapolation method based on the historical lane structure *F*_*t*−1_ from the previous frame *L*_*t*−1_ to perform structural completion. Extract the trajectory direction vector ${\vec{\nu }}_{i}$ from the end of each lane marking, and generate extended points along this direction with a step size of δ for *k* iterations. To extrapolate the lane lines from the previous frame *L*_*t*−1_, first extract the set of terminal points and compute direction vectors as shown below:


$$\begin{array}{l} L_{t-1}=\left\{\left(x_{i},y_{i}\right)|i=1,...,N\right\} \end{array}$$ (5)

$$\begin{array}{l} {\vec{v}}_{i}=\left(x_{i}^{N}-x_{i}^{N-1},y_{i}^{N}-y_{i}^{N-1}\right) \end{array}$$ (6)

where N denotes the serial number of the terminal point of this lane marking line. Project the extended points $\left(x_{i}^{N+j},y_{i}^{N+j}\right)$back onto the image and interpolate them to generate a completion mask *M*_*comp*_. This mask fills in the blurred regions while preserving original information elsewhere, yielding an enhanced $F_{t}^{\prime }$. Subsequently, a CNN network is employed to extract multi-scale features (Pauly et al., 2003; Qin et al., 2020) from $F_{t}^{\prime }$.


$$\begin{array}{l} \left(x_{i}^{N+j},y_{i}^{N+j}\right)=\left(x_{i}^{N}+j\cdot \text{λ}\cdot v_{ix},y_{i}^{N}+j\cdot \text{λ}\cdot v_{iy}\right) \end{array}$$ (7)

where *j* = 1, …, *k*, λ∈[1.0, 2.0] is the scaling factor used to constrain the offset distance. These extrapolated points are projected back to the image space to form a structural completion mask, which is used to reconstruct the blurred areas (see Equation 8).


$$\begin{array}{l} F_{t}^{\prime }=F_{t}\cdot \left(1-M_{\text{blur}}\right)+{\hat{L}}_{t}\cdot M_{\text{blur}} \end{array}$$ (8)

### 3.2 Blur-guided consistency reasoning

To further extract the latent yet non-explicit lane structure information within blurry regions, this paper designs a Blur-Guided Consistency Reasoning Module (BCRM) to identify whether these areas exhibit structural stability. In such regions, if the underlying structure remains stable despite being obscured by motion blur, inter-frame features should demonstrate no significant variations. Based on this, BCRM first models the difference between the structural representations of the current frame and the previous frame as $H_{\text{diff}}\in {\mathbb{R}}^{1\times H\times W}$, which measures the per-pixel disparity between the features of two consecutive frames. To enhance representational capability, structural attention weights α_*c*_ are introduced (Kim et al., 2017; Lin et al., 2021). To quantify the temporal variation in feature representations across frames, we compute the weighted pixel-wise difference between two consecutive frames as:


$${\text{H}}_{\text{diff}}(i,j)=\sum_{c=1}^{C}\alpha_{c}\cdot |{\text{F}}^{\text{'}}(c,i,j)-{\text{F}}_{t-1}(c,i,j)|$$ (9)

$\text{where }\Sigma_{c=1}^{C}\alpha_{c}=1$, represents the learned attention weight for channel c, emphasizing more discriminative features during structural change estimation. The differential heatmap H_diff_ is normalized and interpolated with scaling to obtain the final structural change heatmap ${\text{H}}_{\text{diff}}^{\prime }$.

To determine whether the blurred region contains a stable lane structure, a two-stage inference strategy is adopted, Global Stability Assessment, The structural difference heatmap Hdiff between the current frame and the previous frame is computed according to Equation 9. Based on this, the average structural stability score *S*score within the blur region M_blur_ is calculated using Equation 10, which quantifies the overall temporal consistency of the region. Pixel-wise Stable Mask Generation, If the global score satisfies *S*_score_>δ, the region is considered structurally reliable, and pixel-level refinement is performed to generate the final stable region mask. Otherwise, if *S*_score_ ≤ δ, the region is assumed to have experienced significant scene changes (e.g., occlusion or object insertion), and the stable region mask M_stable_ is assigned as an all-zero matrix to suppress unreliable structural completion.

The two-stage inference logic is consolidated into a single, concise mathematical expression. We employ the Iverson Bracket notation, [*P*], which evaluates to 1 if the condition P is true, and 0 otherwise. The final stable mask, M_stable_, is generated by multiplying the outcomes of the global stability assessment and the pixel-wise local variation check. This ensures that a pixel is only marked as stable if all conditions are met simultaneously, as shown in Equation 11.


$$\begin{array}{l} S_{score}=\frac{\underset{i,j}{\sum }\left(1-{\text{H}}_{diff}\left(i,j\right)\right)\cdot {\text{M}}_{blur}\left(i,j\right)}{\underset{i,j}{\sum }{\text{M}}_{blur}\left(i,j\right)} \end{array}$$ (10)

$$\begin{array}{l} {\text{M}}_{stable}\left(i,j\right)=\left[S_{score}>\delta \right]\cdot {\text{M}}_{blur}\left(i,j\right)\cdot [{\text{H}}_{diff}\left(i,j\right)<\epsilon s] \end{array}$$ (11)

where ϵ denotes the threshold for intensity of variation, which is determined based on the statistical distribution of inter-frame feature differences within non-blurred lane regions across the training set. Stable lane structures should exhibit only minor fluctuations between consecutive frames, whereas significant deviations are more likely attributable to occlusion or illumination changes. Specifically, the mean μ and standard deviation σ of Hdiff values in stable regions are computed, with ϵ set to μ + k σ (*k* = 0.7).

### 3.3 Blur-temporal modeling Mamba

**Figure 3:**
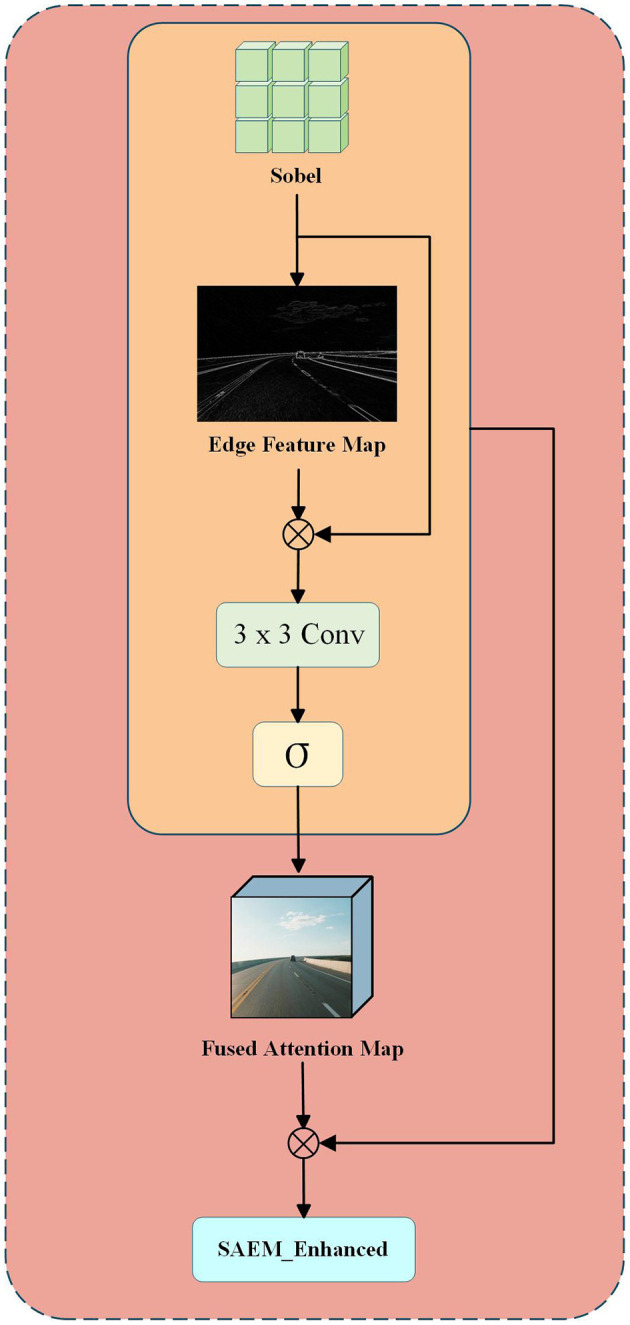
Enhancement of lane marking edge details via Sobel-based edge extraction, structural attention mapping, and feature fusion.

In the field of image processing, high-frequency operators are frequently employed to enhance edge feature information within images (see Figure 3). Images typically contain characteristics across both high and low frequencies. Low-frequency features encompass global structures and color information, whereas high-frequency components primarily consist of edges and fine details that distinctly delineate the contours of target objects. For lane detection tasks, edge contour features hold greater significance than color distribution patterns. Accordingly, this paper introduces the Sobel high-frequency operator to extract edge guidance and designs a structural attention module for enhancement purposes, thereby compensating for detail losses incurred during preprocessing stages due to redundant frame removal (Kanopoulos et al., 1988). Specifically, for an image feature F∈ℝ^*C*×*H*×*W*^, it is first converted to grayscale and then processed with Sobel filtering to extract edge feature maps. Subsequently, residual convolution combined with Sigmoid activation is employed to generate the structural attention map $A_{\text{Struct}}\in {\left[0,1\right]}^{C\times H\times W}$. Then, a structural attention map is generated through a convolutional layer, as detailed below:


$$\begin{array}{l} {\text{E}}_{\text{D}}=\text{Sobel}\left(\text{Gray}\left(\text{F}\right)\right) \end{array}$$ (12)

$$\begin{array}{l} {\text{A}}_{\text{Struct}}=\text{σ}\left({\text{Conv}}_{3\times 3}\left({\text{E}}_{\text{D}}\right)\right) \end{array}$$ (13)

Perform a channel-wise Hadamard product between the attention map and the original feature map to obtain an enhanced feature map F_SAEM_(*c, i, j*) with prominent edge structures while preserving original semantic information. To integrate supervision signals from structurally stable regions, utilize the structural stability mask ${\text{M}}_{\text{stable}}\in {\mathbb{R}}^{\left\{1\times H\times W\right\}}$generated by the BCRM module as a weight map for explicit feature fusion, yielding deep feature embeddings ${\text{F}}_{\text{SAF}}\in {\mathbb{R}}^{C^{\prime }\times H\times W}$.


$$\begin{array}{l} {\text{F}}_{\text{SAF}}\left(c,i,j\right)={\text{F}}_{\text{SAEM}}\left(c,i,j\right)\cdot \left(1+\text{λ}\cdot {\text{M}}_{\text{stable}}\left(i,j\right)\right) \end{array}$$ (14)

where λ denotes the fusion weight coefficient, governing the degree of structural saliency enhancement.

**Figure 4:**
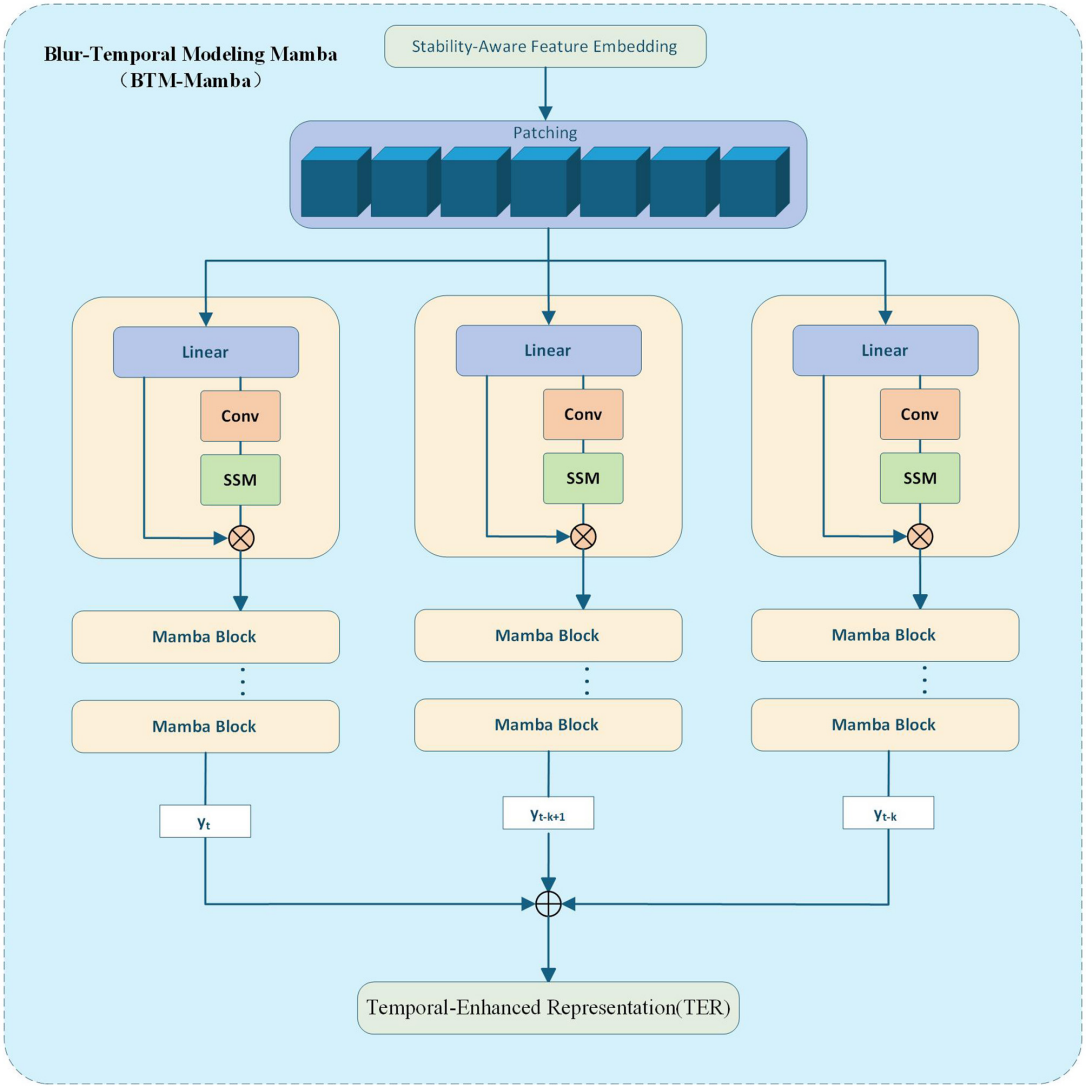
Depth features are stabilized, sequenced, modeled via parallel Mamba Blocks, and reconstructed into a temporal-enhanced representation (TER).

The overall modeling pipeline for temporal feature enhancement using the Mamba architecture is illustrated in Figure 4, which includes patching, long-range modeling, and unpatching to obtain final temporal-enhanced representations (TER). This figure visually explains how the StaBle-MambaNet encodes vertical spatial continuity and inter-frame dynamics. To fully capture the structural temporal evolution characteristics of ambiguous regions, this paper employs the Mamba state space modeler to model the fused features. The Mamba network effectively models long-range dependencies across frames through its selective state space (SSM) architecture, thereby enhancing the model features of lane markings (Patro and Agneeswaran, 2025).

Specifically, we flatten the deep feature zzzzs embedded in F_SAF_ row-wise, treating each row's feature vector as a temporal token to form a sequence of length H fed into the Mamba network. This approach preserves spatial continuity along the vertical dimension and facilitates the model's capture of top-down structural evolution in lane markings. ${𝔭}_{i}\in {\mathbb{R}}^{d}$ denotes a temporal token in the sequence of feature vectors by Equation 15.


$$\begin{array}{l} \left\{{\text{p}}_{1},{\text{p}}_{2},...,{\text{p}}_{N}\right\}=\text{Patch}\left({\text{F}}_{\text{SAF}}\right) \end{array}$$ (15)

The patch sequence is fed into stacked Mamba blocks, wherein Mamba employs a state-space recurrent formulation to model sequence dependencies ${\text{h}}_{t}\in {\mathbb{R}}^{d}$ (see Equation 16).


$$\begin{array}{l} h_{t}=A\left({\text{x}}_{t}\right)\cdot {\text{h}}_{t-1}+B\left({\text{x}}_{t}\right) \end{array}$$ (16)

Unlike traditional SSMs, the parameters *A*(·)and *B*(·)in Mamba are input-dependent functions. By employing a sliding window mechanism combined with gating mechanisms, it processes token sequences in parallel, thereby enhancing its capability to model dynamically varying inputs and improving long-range dependency awareness. For {h_1_, h_2_, …, h_*N*_}, to obtain globally unified representational features, this paper maps them back to the original feature map spatial distribution. F_TER_ preserves both the temporal evolution characteristics of each local structure and maintains spatial contextual alignment(see Equation 17).


$$\begin{array}{l} {\text{F}}_{\text{TER}}=\text{Unpatching}\left({\text{h}}_{1},{\text{h}}_{2},...,{\text{h}}_{N}\right)\in {\mathbb{R}}^{C\times H\times W} \end{array}$$ (17)

### 3.4 Structural supervision and multi-objective learning

In the collected data, due to the angular limitations of acquisition equipment, lane markings rarely appear perfectly vertical or horizontal in images. Typically, they exhibit an inclined configuration within captured frames (Liu et al., 2017). To ensure the model preserves structural coherence of lane markings under blurry frames or occlusion scenarios, we incorporate inter-frame structural consistency loss, which models the structural discrepancy between temporally enhanced features ${\text{F}}_{\text{TER}}^{\left(t\right)}$ and ${\text{F}}_{\text{TER}}^{\left(t-1\right)}$ across consecutive frames. According to Equation 18, the inter-frame structural consistency loss quantifies the structural discrepancy between temporally enhanced features ${\text{F}}_{\text{TER}}^{t}$ and ${\text{F}}_{\text{TER}}^{t-1}$, ensuring temporal coherence in lane structure modeling under blurred conditions.


$$\begin{array}{ll} L_{cons} & =\frac{1}{\sum M_{stable}}\sum_{i,j}M_{stable}\left(i,j\right)\cdot ∥F_{TER}\left(t\right)\left(i,j\right) \end{array}$$ (18)

$$\begin{array}{l} \;-F_{TER}\left(t-1\right)\left(i,j\right){∥}_{1} \end{array}$$ (19)

where ||·||_1_ denotes the L1 norm, which measures the structural representation discrepancy between two frames at corresponding spatial locations. This loss function effectively enforces spatial consistency of lane structures across consecutive frames, preventing abrupt structural transitions caused by motion blur or transient occlusion.

To more accurately measure the overlap between predicted lanes and ground-truth lanes while ensuring alignment between optimization objectives and evaluation criteria, this paper introduces a slope-aware LaneIoU method that incorporates consideration of lane width and add directionally adaptive 'virtual lane widths' for each point prediction to simulate human visual perception of angular variations in lane markings, thereby further adjusting the virtual width of lane lines. The formula for virtual width setting is given by Equation 19, where $\text{Δ}x_{n}^{k}$ and $\text{Δ}y_{n}^{k}$ represent the gradient variations of the k-th predicted lane marking at the n-th sampling point, indicating the local changes within the n-th row that reflect its slope steepness.


$$\begin{array}{l} w_{n}^{k}=\frac{w_{lane}}{2}\cdot \frac{\sqrt{{\left(\text{Δ}x_{n}^{k}\right)}^{2}+{\left(\text{Δ}y_{n}^{k}\right)}^{2}}}{\text{Δ}y_{n}^{k}} \end{array}$$ (20)

When the lane is in a vertical state, $\text{Δ}x_{n}^{k}=0$; its width equals the actual width *W*_*lane*_/2 of the lane markings. However, when inclined, the width increases with the angle of inclination. Finally, the calculation formula for LaneIOU is given by Equation 20.


$$\begin{array}{l} LaneIOU=\frac{\sum_{n=0}^{H}I_{n}}{\sum_{n=0}^{H}U_{n}} \end{array}$$ (21)

where, *I*_*n*_ and *U*_*n*_ denote the intersection and union between predicted and ground-truth lanes in the nth row of pixels, respectively, enabling area estimation through incorporation of virtual width.

To achieve end-to-end optimization of the model, the final training objective function integrates four supervisory signals, forming a jointly optimized multi-task learning framework. It comprises: a classification loss L_*cls*_ for determining the presence of lane markings; a regression loss L_*xytl*_ for precise localization of lane coordinates; a structure-aware slope-adaptive LaneIoU loss L_*LaneIoU*_; and a temporal consistency loss L_*cons*_ serving as a regularization term to ensure structural continuity in ambiguous scenarios. Additionally, a temporal consistency loss term is incorporated as a regularization constraint to formulate a joint optimization framework.


$$\begin{array}{ll} L_{total} & ={\text{λ}}_{cls}L_{cls}+{\text{λ}}_{xytl}L_{xytl} \end{array}$$ (22)

$$\begin{array}{l} \;+{\text{λ}}_{LaneIOU}L_{LaneIOU}+{\text{λ}}_{cons}L_{cons} \end{array}$$ (23)

whereλ_*cls*_ = 2, λ_*xytl*_ = 0.2, λ_*IOU*_ = 2, L_*cls*_ denotes the binary cross-entropy (BCE) loss for lane existence prediction, L_*reg*_ represents the coordinate regression loss of lane points (x,y), L_*LaneIoU*_ signifies the slope-aware LaneIoU loss, and L_*cons*_ corresponds to the structural consistency loss After comprehensive consideration of all factors, the complete algorithm proposed in this paper is presented as Algorithm 1.

Algorithm 1 **StaBle-MambaNet.**

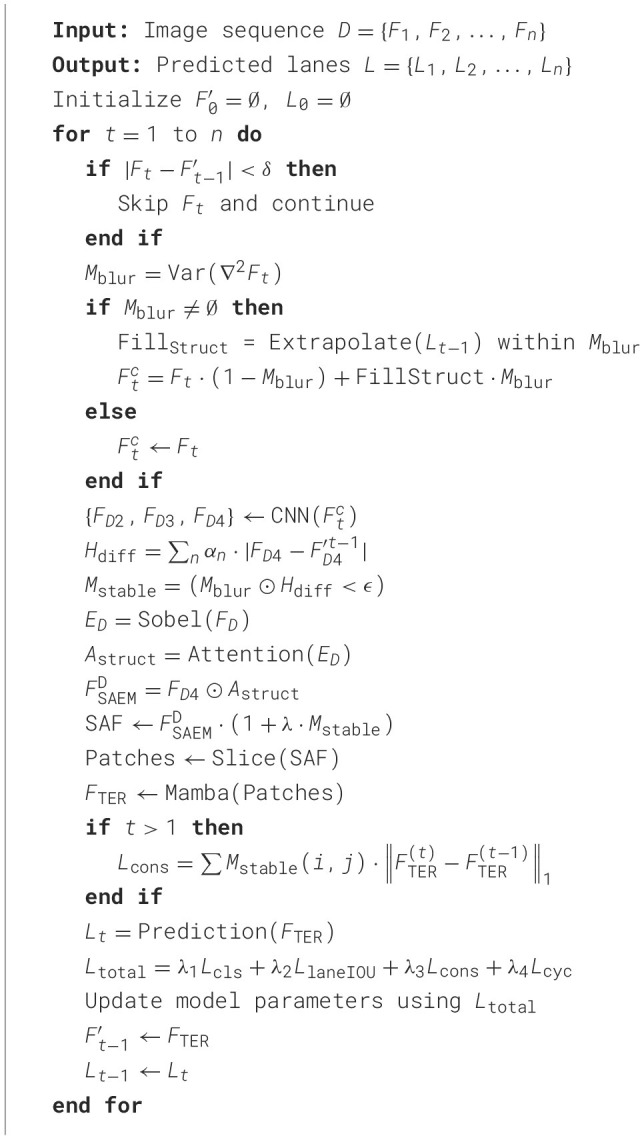

## 4 Experiment results

### 4.1 Experimental set-up

The methods proposed throughout this research were validated and analyzed both during the training process and testing phase. To ensure experimental efficiency and result reproducibility, all relevant experiments were conducted under a Linux operating system environment. The experimental platform specifications are as follows: processor–AMD Ryzen 9 7950X with 16 cores at 4.5GHz; graphics card–GeForce RTX 4090 (24GB VRAM); CUDA version 11.8; programming language–Python 3.10. During the experiments in this chapter, the AdamW optimizer was employed for gradient descent optimization with a total of 100 training epochs. For image preprocessing, pixel values were standardized to mitigate variations across different images. A cosine annealing schedule was applied for learning rate decay during training, with an initial learning rate (lr) set at 0.0006 and a batch size configured to 24.

This paper conducts experiments using the CULane dataset and the CurveLanes dataset. The CULane dataset contains over 133,000 frames captured in urban driving scenarios, with diverse environmental conditions including normal illumination, crowded traffic, nighttime scenes, shadows, and lane disappearance. It emphasizes large-scale evaluation across challenging factors such as occlusion by vehicles and strong lighting variations. In contrast, the CurveLanes dataset specifically focuses on road geometries with frequent curves and slope variations, comprising approximately 150,000 frames with higher coverage of complex lane topologies and long-range curvature. Compared with CULane, CurveLanes places stronger emphasis on curved-road scenarios and structural continuity under geometric deformation. These complementary characteristics make the two datasets jointly suitable for assessing both robustness to environmental degradation and adaptability to road shape complexity, thereby providing a comprehensive validation of the proposed approach.

For reproducibility, the key hyperparameters used in modules are detailed in Table 1.

**Table 1:** Hyper-parameter list.

**Param**.	**Module**	**Description**	**Value**
δ	SARM	Laplacian variance blur threshold	100
*K*	SARM	Iterations for directional lane extrapolation	5
λ	SARM	Scaling factor for extrapolation step	1.5
δ	BCRM	Threshold for global stability score	0.85
ε	BCRM	Threshold for local feature variation	0.1
λ′	BTM-Mamba	Weight for fusing stability mask with features	1.0
λ_cons_	SSML	Loss weight for temporal structural consistency	0.1
λ_cls_	SSML	Loss weight for lane existence classification	2.0
λ_xytl_	SSML	Loss weight for lane coordinate regression	0.2
λ_IOU_	SSML	Loss weight for slope-aware LaneIoU	2.0

### 4.2 Experimental results and analysis

This chapter first conducts training on CULane to evaluate the detection performance of the proposed method. Subsequently, validation is performed on the newly introduced CurveLanes dataset to assess the generalization capability of the model StaBle-MambaNet. Finally, ablation experiments are carried out for each component to verify the rationality of the overall framework and individual component designs. To validate the effectiveness of the lane detection framework StaBle-MambaNet proposed in this chapter, it was trained on the public dataset CULane with subsequent analysis of results. Among these, ResNet34 and DLANet34 were selected as backbone networks.

Experimental results on the CULane dataset demonstrate that the proposed method StaBle-MambaNet achieves superior performance across multiple scenarios (see Table 2). Overall, compared with existing approaches, StaBle-MambaNet maintains high F1 scores in diverse driving conditions. Notably, it exhibits excellent performance in challenging environments such as nighttime illumination, curved roads, shadow obstructions, and lane disappearance–conditions prone to causing blurred lane images–further demonstrating its adaptability to varying scenarios and highlighting its practical application value.

**Table 2:** F1 score comparison on the CULane dataset and other mainstream methods.

**Method**	**Backbone**	**Normal**	**Congested**	**Bright**	**Shadow**	**Wireless**	**Arrow**	**Curve**	**Crossroads**	**Nighttime**	**Total**
RESA	ResNet34	91.9	72.4	66.5	72	46.3	88.1	68.6	1896	69.8	74.5
UFLD	ResNet18	87.7	66	58.4	62.8	40.2	81	57.9	1743	62.1	68.4
UFLD	ResNet34	90.7	70.2	59.5	69.3	44.4	85.7	69.5	2037	66.7	72.3
UFLDv2	ResNet18	91.8	73.3	65.3	75.1	47.6	87.9	68.5	2075	70.7	75
UFLDv2	ResNet34	92.5	74.8	65.5	75.5	49.2	88.8	70.1	1910	70.8	76
LaneATT	ResNet18	91.17	72.71	65.82	68.03	49.13	87.82	63.75	**1020**	68.5	75.13
LaneATT	ResNet34	92.14	75.03	66.47	78.15	49.39	88.38	67.72	1330	70.72	76.68
CondLaneNet	ResNet18	92.87	75.79	70.72	80.01	52.39	89.37	72.4	1364	73.23	78.14
CondLaneNet	ResNet34	93.38	77.14	71.17	79.93	51.85	89.89	**73.88**	1387	73.92	78.74
CLRNet	ResNet18	93.11	77.51	73.32	80.83	51.75	90.44	68.11	1072	74.61	79.18
CLRNet	ResNet34	93.62	78	72.51	81.4	53.11	90.51	71.43	1244	75.54	79.73
StaBle-Net	ResNet34	93.75	78.2	**72.89**	81.75	53.45	90.72	71.98	1084	75.89	80.12
StaBle-Net	DLA34	**93.82**	**78.45**	73.12	**82.1**	**53.78**	**90.89**	72.3	1147	**76.02**	**80.53**

Bold values indicate the best performance of that metric among the methods.

Based on the selected backbone network versions, the medium-sized ResNet34 variant of StaBle-MambaNet demonstrates superior performance across all metrics compared to mainstream anchor-based detection models using the same ResNet34 architecture. For instance, when benchmarked against CondLaneNet (Liu L. et al., 2021), StaBle-MambaNet achieves a 1.38 percentage point improvement in F1 score while exhibiting varying degrees of performance enhancement across diverse scenarios. Furthermore, employing DLANet34 (Su et al., 2022) as the backbone network elevates the overall model performance, attaining an optimal F1 score of 80.53. Notably, it successfully leverages inter-frame temporal dynamics to enhance robustness in challenging conditions such as nighttime illumination, shadows, and wireless interference–demonstrating broad environmental adaptability and providing a more stable solution for lane marking detection during daily driving operations.

Comparative analysis with different anchor-based lane detection algorithms reveals inherent limitations in UFLD and UFLDv2 due to architectural constraints, resulting in suboptimal performance under complex scenarios. Although recent high-performing anchor-based methods like CondLaneNet and CLRNet exhibit commendable detection capabilities across multiple contexts, StaBle-MambaNet consistently outperforms them in various settings. Particularly in environments with significant lighting variations (e.g. nighttime, glare, shadows), where multiple factors adversely affect data quality, StaBle-MambaNet maintains superior performance stability–further validating its feasibility and application potential.

Experimental results on the CULane dataset confirm that StaBle-MambaNet sustains high detection accuracy across diverse scenarios while demonstrating enhanced generalization capability in complex situations. When configured with DLA34 as the backbone network, global feature extraction capacity is significantly strengthened, enabling higher overall detection precision. These advantages maximize StaBle-MambaNet's potential for rapid driving scenarios.

To further validate generalization capabilities, evaluation was conducted on the CurveLanes dataset (see Table 3). The proposed method achieves favorable performance across F1 score, precision, and recall metrics. Compared to state-of-the-art networks from recent years (CondLane and CLRNet), StaBle-MambaNet reaches an F1 score of 86.34. While not achieving peak values in precision/recall individually, these metrics remain balanced within acceptable ranges. Specifically, the recall rate of 83.29 substantiates StaBle-MambaNet's exceptional performance and practical utility for lane detection tasks–particularly its cross-dataset applicability and robustness under complex environmental conditions.

**Table 3:** Performance comparison on the CurveLanes dataset with different backbones, showing F1 score, accuracy, and recall.

**Method**	**Backbone network**	**F1**	**Accuracy**	**Recall**
SCNN	VGG16	65.02	76.13	56.74
CondLane	ResNet18	85.09	87.75	82.58
CondLane	ResNet34	85.92	88.29	**83.68**
CLRNet	DLANet34	86.1	**91.4**	81.3
StaBle-Net	DLANet34	**86.34**	89.5	83.29

Bold values indicate the best performance of that metric among the methods.

Compared to CondLane with ResNet34 architecture, StaBle-MambaNet achieves a 0.32 percentage point increase in F1 score and an improvement of 1.21 in precision, albeit at the cost of a 0.39 decrease in recall. These experimental results demonstrate that StaBle-MambaNet can detect more lane markings under curved road scenarios. While CLRNet based on DLANet34 exhibits higher precision, its relatively low recall rate of 81.30 suggests potential missed detections in curved environments. In contrast, the DLANet34 variant of StaBle-MambaNet successfully addresses this issue through feature enhancement strategies leveraging inter-frame temporal dynamics, thereby ensuring improved recall on CurveLanes while maintaining high precision at 89.50. The experimental findings indicate that StaBle-MambaNet effectively reduces false positive detections while maintaining accurate lane identification.

**Figure 5:**
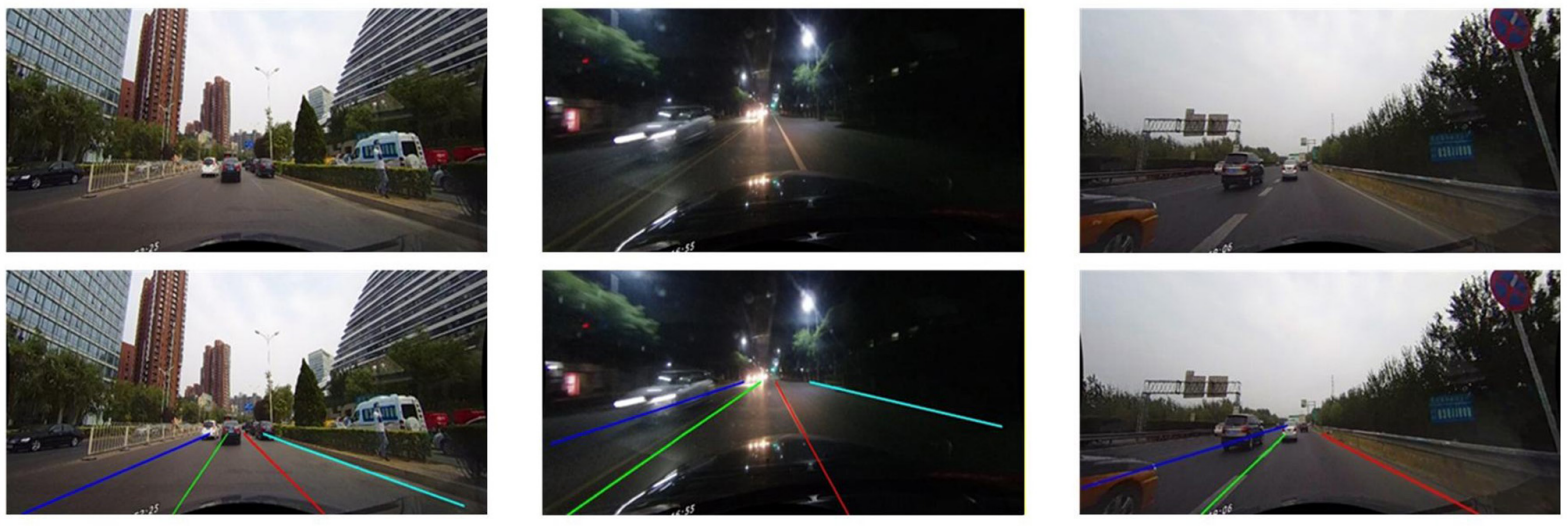
Visualization of lane detection results under various data conditions (images reproduced with permission from the CULane dataset, https://xingangpan.github.io/projects/CULane.html), licensed under CC-BY-NC.

Overall performance metrics across F1 score, precision, and recall on the CurveLanes dataset confirm StaBle-MambaNet's superior efficacy. Its feature enhancement approach enables robust lane recognition in complex scenes, enhancing detection reliability and practical deployment feasibility. Figure 5 presents qualitative detection results across three representative scenarios: On the left is an image of normal quality, in the center is a low-resolution image, and on the right is an image captured under blurry scene conditions. As shown in the Figure 5, StaBle-MambaNet consistently maintains lane detection accuracy and avoids false positives, even under visually degraded conditions. Visual analysis reveals consistent lane detection capability without false positives or missed detections even under suboptimal image quality conditions.

This paper compares the performance in terms of accuracy and inference time between the two-stage cascade approach “global restoration followed by lane detection” and Stable-MambaNet. Using CLAHE and DeblurGAN as preprocessing techniques respectively, both are connected to the same detection head (Baseline). Additionally, an ablation study is conducted on the SARM and BCRM modules of Stable-MambaNet using Baseline as reference.

**Figure 6:**
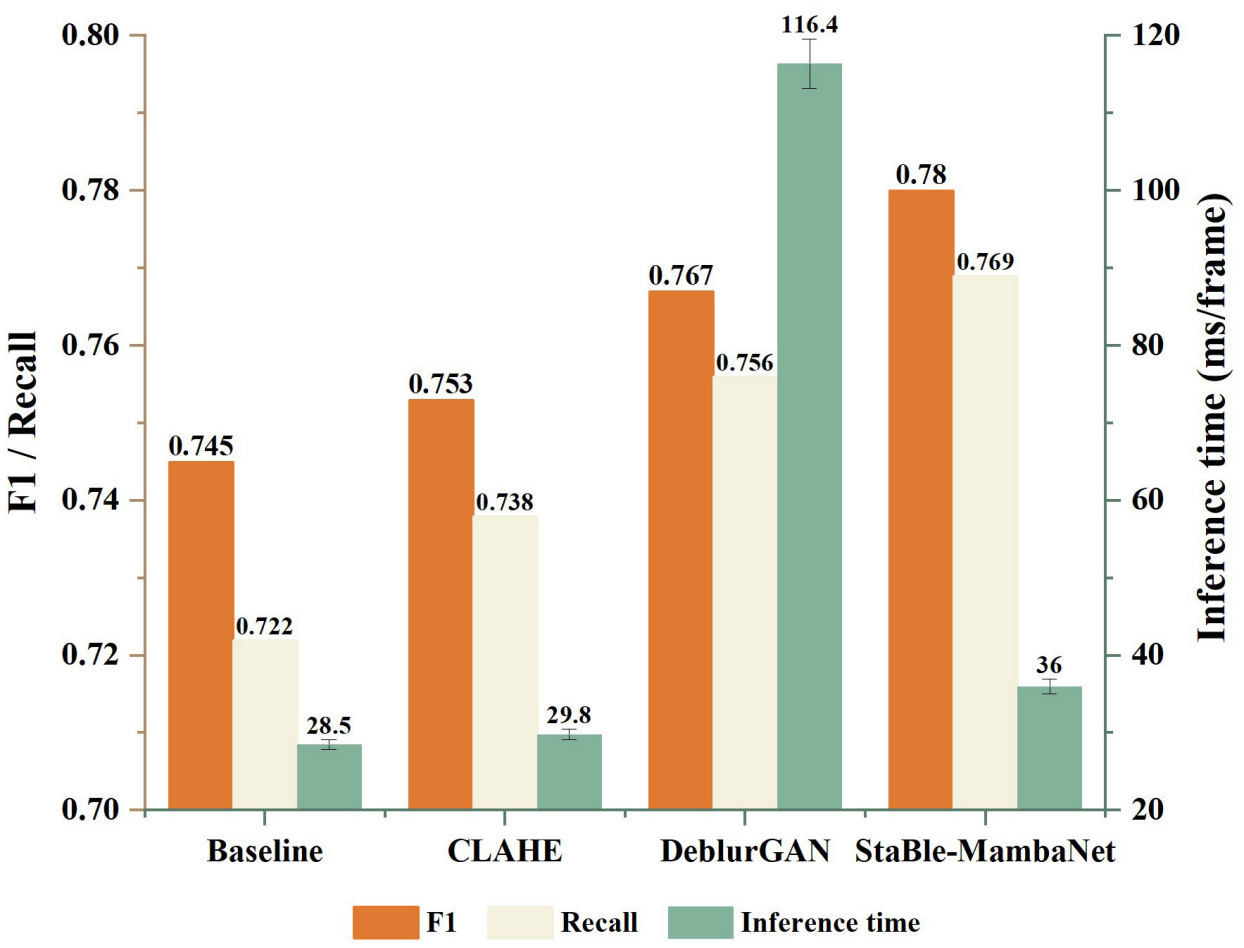
Comparison of performance and efficiency across four scenarios.

This paper compares the performance in terms of accuracy and inference time between the two-stage cascade approach “global restoration followed by lane detection” and Stable-MambaNet. Using CLAHE and DeblurGAN as preprocessing techniques respectively, both are connected to the same detection head (Baseline). For DeblurGAN, to ensure fairness, the network was not fine-tuned on the lane detection dataset but instead employed directly as a preprocessing module for the baseline detector. Inference was performed on the same hardware platform described in Section 4.1. During testing, the model operated with a batch size of 1 and utilized default optimization settings. Additionally, an ablation study is conducted on the SARM and BCRM modules of Stable-MambaNet using Baseline as reference. The results are illustrated in Figure 6. Regarding precision metrics, the CLAHE-based cascade yields only marginal improvements over the baseline: F1 increases from 0.745 to 0.753 while recall rises from 0.722 to 0.738, demonstrating limited utility of simple contrast enhancement for structural recognition. With the introduction of DeblurGAN, F1 further improves to 0.767 (recall = 0.756), indicating that global restoration can compensate for certain information losses caused by blurring. However, this accuracy gain comes at the cost of significantly increased inference time, reaching 116.4 ms/frame, which fails to meet real-time application requirements. In contrast, Stable-MambaNet achieves an F1 score of 0.780 and recall of 0.769 without global restoration, maintaining an inference time of merely 36 ms/frame. Compared to the DeblurGAN cascade, it delivers superior accuracy with reduced latency; relative to the CLAHE cascade, it exhibits substantial precision advantages while introducing only slightly higher delay than the baseline.

**Figure 7:**
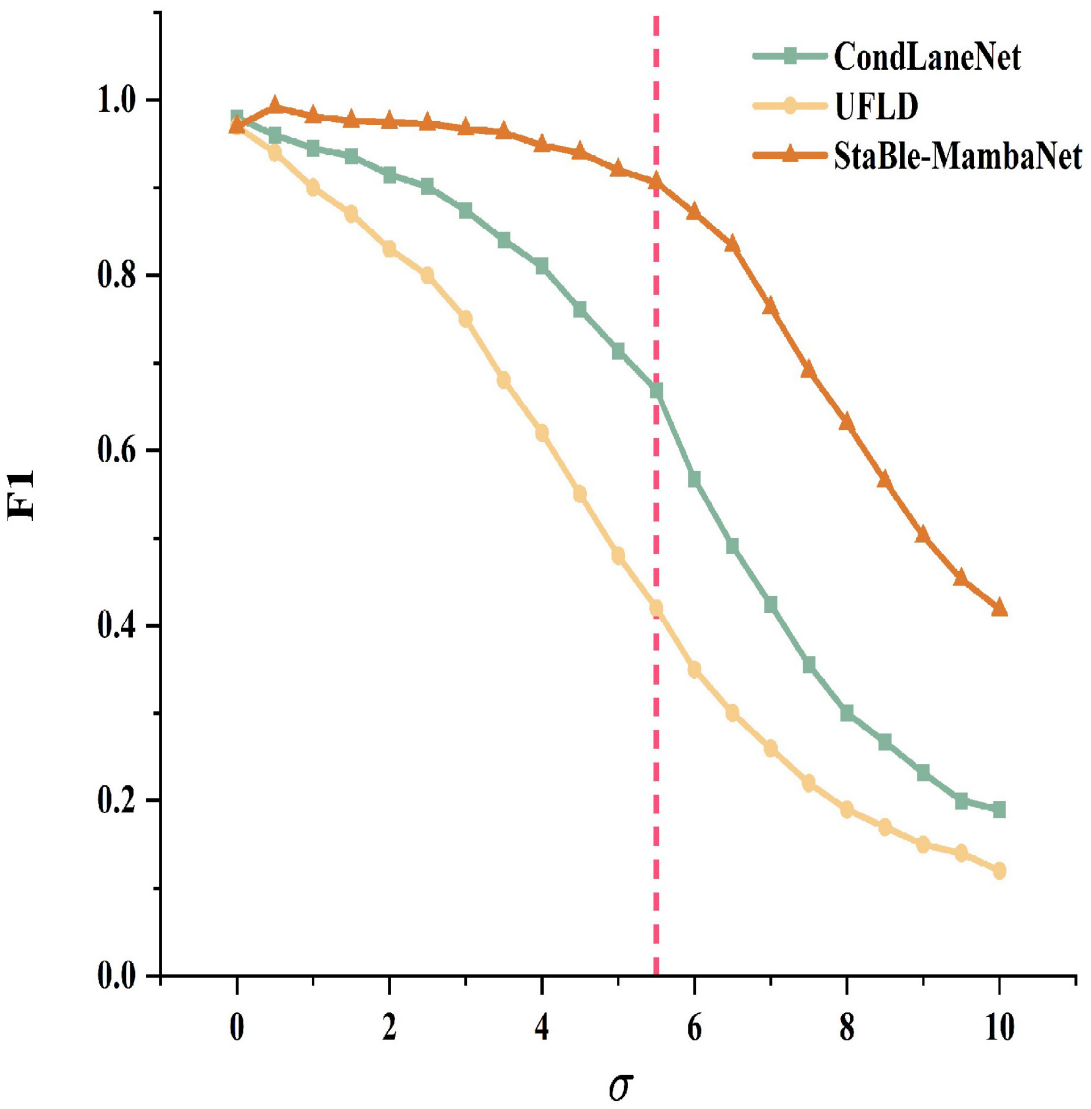
Robustness comparison under varying Gaussian blur σ levels.

To simulate varying degrees of local information loss from mild to severe conditions, Gaussian blur with standard deviation σ was applied to lane marking regions. The degradation behaviors of Stable-MambaNet, CondLaneNet, and UFLD were evaluated through F1-σ curves as shown in Figure 7. Under mild blurring (σ ≤ 2), all three methods achieve near-perfect F1 scores with negligible differences. When σ≈3, CondLaneNet begins performance decline, whereas UFLD enters an earlier decay phase and maintains consistently lower performance throughout. At an intermediate blur intensity of approximately σ≈5.5, this threshold is demarcated by a vertical dashed line to indicate the critical point where performance disparities become amplified; furthermore, all methods exhibit an accelerating trend in their performance degradation. Under heavy blurring (σ>6), all approaches experience further degradation, yet Stable-MambaNet demonstrates a gentler slope of decline, preserving its maximum F1 advantage across a wider range. Overall, the proposed method exhibits more stable performance degradation trajectories across the entire spectrum of blur intensities, reflecting enhanced resilience to local information loss.

**Figure 8:**
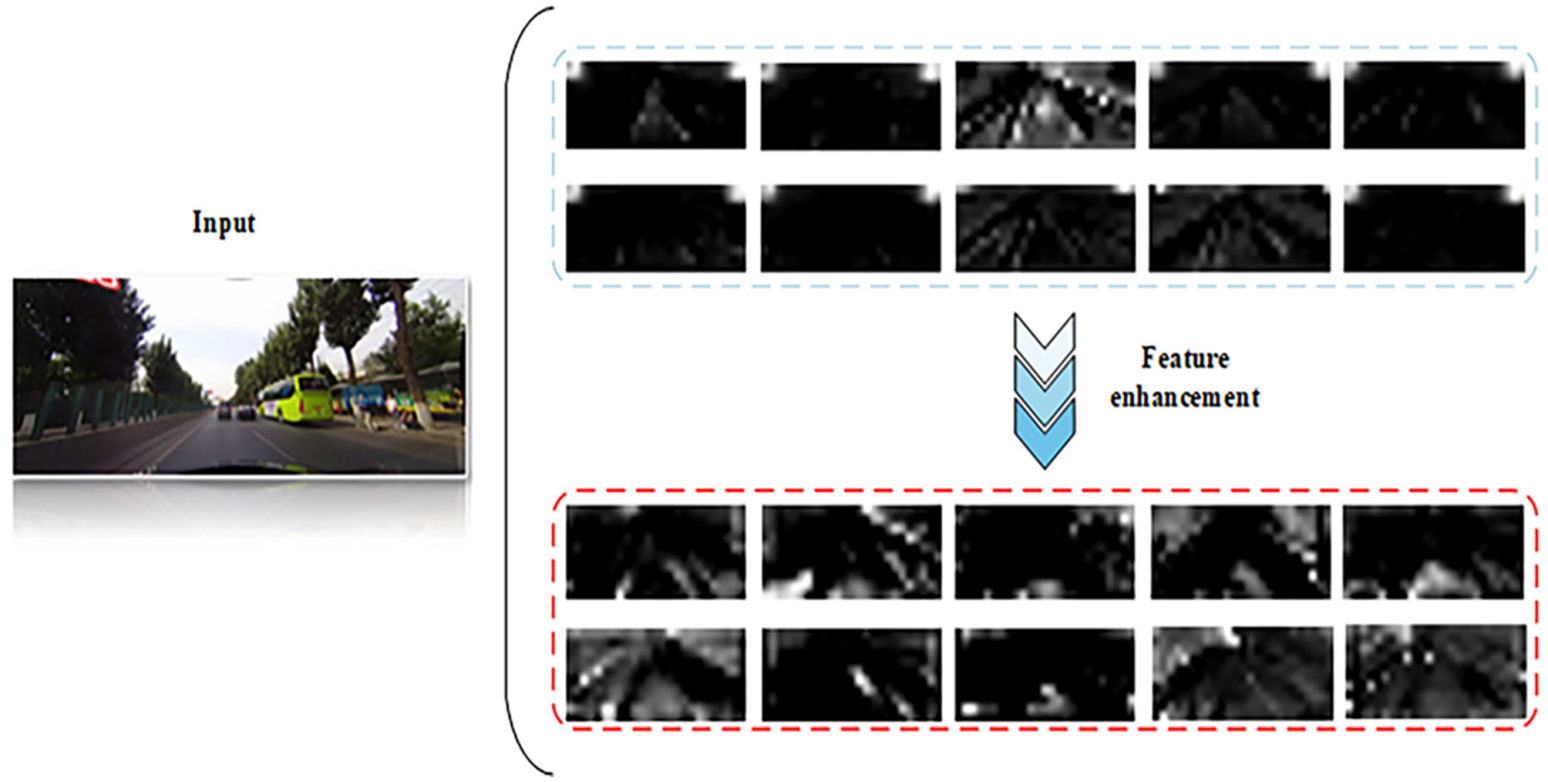
Visualization of Feature Maps Before and After Enhancement, (reproduced with permission from the CULane dataset, https://xingangpan.github.io/projects/CULane.html), licensed under CC-BY-NC.

As illustrated in Figure 8, the visualization results of the top 10 channel feature maps after processing by the SARM module and BTM-Mamba in StaBle-MambaNet are presented. The original channels exhibit the baseline model's corresponding layer feature map visualization, while the processed channels demonstrate enhanced features characterized by sharper boundary focus and improved lane separability. Observations indicate that the augmented feature maps exhibit heightened attention to region-specific characteristics relevant to lane markings, with significantly enhanced contrast between lane markings and background regions. This observation further validates the effectiveness of the proposed feature enhancement scheme.

### 4.3 Ablation experiment

Evaluate the contribution values of each component's functionality within the lane detection framework StaBle-MambaNet proposed in this chapter. Table 4 demonstrates the impact of different components in StaBle-MambaNet on the final model performance. Throughout the ablation study, StaBle-MambaNet employs ResNet34 as its backbone architecture and conducts experiments on the CULane dataset. The experimental results presented in Table 4 indicate that incorporating each component yields positive effects on the F1 score–a key detection metric for StaBle-MambaNet–with optimal overall model performance achieved when all modules are utilized collectively. Baseline model: without incorporating the SARM and BTM-Mamba modules, the baseline model achieved an F1 score of 79.73.

**Table 4:** Ablation study.

**Baseline**	**SARM**	**BTM-Mamba**	**SSML**	**F1**
✓				79.73
	✓			79.88
		✓		79.96
			✓	79.77
	✓		✓	79.99
	✓		✓	80.05
		✓	✓	80.00
	✓	✓	✓	**80.12**

Bold values indicate the best performance of that metric among the methods.

Only SARM: when only the SARM was added, the F1 score increased to 79.88, demonstrating that SARM effectively restores and enhances edge features, enabling the model to focus more on detailed characteristics and thereby improving detection capability. Only BTM-Mamba module: upon adding only the BTM-Mamba module, the F1 score further rose to 79.96. This indicates that BTM-Mamba plays a positive role in global information modeling and long-range feature enhancement. Concurrently, fused features containing ambiguous information effectively guide the Mamba network to attend to trustworthy regions, enhancing lane line detection performance. This module critically contributes to accurate lane localization, particularly improving the model's long-term tracking ability for lane lines in high-speed driving scenarios. Only SSML: after solely optimizing SSML, the F1 score ascended to 79.77, showing that joint optimization of lane classification loss, position regression loss, and structure-aware LaneIoU loss enhances the model's accurate performance evaluation. Any Two-Module Synergy: Any combination of SSML with either BTM-Mamba or SARM outperformed individual module additions, further confirming each module's positive impact on StaBle-MambaNet performance.

With all modules fully integrated, the F1 score reached 80.12–an increase of 0.39 percentage points from the baseline and superior to using any single component alone. This demonstrates that collective interaction among modules effectively enhances the model's feature learning capacity, leading to improved performance in complex scenes. These results indicate stronger adaptability of this approach across diverse environmental conditions for lane detection tasks.

In summary, both SARM, BTM-Mamba, and SSML individually improve model performance. However, their synergistic effect further enhances feature representation of lane lines during detection. This ablation study validates the effectiveness of the proposed method in this chapter.

## 5 Discussion

To address the challenge of degraded lane detection performance caused by motion blur in autonomous driving scenarios such as high speeds and complex lighting conditions, this paper proposes a method named StaBle-MambaNet, which employs structural verification within blurred regions and completion guidance for lane detection. Departing from conventional approaches that prioritize global image restoration followed by detection, our method innovatively focuses on determining whether stable lane structures exist within localized blurred areas. Based on this judgment, it performs selective structural completion and temporal modeling to enhance detection robustness and efficiency.

Experimental results demonstrate that StaBle-MambaNet achieves superior performance on public datasets including CULane and CurveLanes, particularly excelling in challenging scenarios prone to blur–such as nighttime conditions, shadows, and curves–where key metrics like F1 score surpass those of multiple state-of-the-art methods. Ablation studies further validate the effectiveness of each innovative module within the framework. In summary, the proposed approach offers an effective and reliable new solution for lane detection under blurry conditions.

Although the proposed method has achieved promising results, there remains room for further optimization. Future research could proceed from the following aspects:

Exploration of Multimodal Fusion Strategies: This study relies exclusively on visual information; however, under extreme motion blur or severe sensor contamination conditions, image-based reconstruction alone may fail to recover structural details. Subsequent efforts should investigate integrating LiDAR or Radar data–sensor modalities exhibiting stronger robustness against illumination variations and adverse weather conditions. Such geometric priors from active sensors can provide reliable structural constraints for ambiguous visual regions, enabling more robust model inference and completion.

Lightweight Deployment for Edge Devices: Despite Mamba's superior efficiency over conventional Transformers, the computational complexity of the entire architecture still poses challenges for resource-constrained in-vehicle computing platforms. The selective state-space formulation of Mamba inherently supports linear-time sequence modeling with lower memory consumption, providing a structural advantage for lightweight adaptation. Future work will explore these directions: (i) knowledge distillation from the full StaBle-MambaNet to compact student models while retaining blur-specific reasoning capability, (ii) structured pruning guided by the stability mask to remove redundant feature channels outside blur-critical regions, and (iii) low-bit quantization of the Mamba blocks to further reduce memory footprint and computational latency.

## Data Availability

Publicly available datasets were analyzed in this study. This data can be found at: https://github.com/SoulmateB/CurveLanes; https://xingangpan.github.io/projects/CULane.

## References

[B1] Bansal R. Raj G. Choudhury T. (2016). “Blur image detection using Laplacian operator and Open-Cv,” in 2016 International Conference System Modeling & *Advancement in Research Trends (SMART)* (Moradabad: IEEE), 63–67. doi: 10.1109/SYSMART.2016.7894491

[B2] Bansal S. Sreeharish A. MadhavaPrasath J. Manikandan S. Madisetty S. Rehman M. Z. U. . (2024). A comprehensive survey of Mamba architectures for medical image analysis: classification, segmentation, restoration and beyond. arXiv [preprint]. arXiv:2410.02362. doi: 10.48550/arXiv.2410.02362

[B3] Chai Y. Wang S. Zhang Z. (2024). A fast and accurate lane detection method based on row anchor and transformer structure. Sensors 24:2116. doi: 10.3390/s24072116 38610330 PMC11014103

[B4] Dong X. Lan J. (2024). Optimal path for automated pedestrian detection: image deblurring algorithm based on generative adversarial network. J. Meas. Eng.12, 298–311. doi: 10.21595/jme.2023.23765

[B5] Gao L. Lu Y. (2025). Research on anchor-based lane line detection algorithm,” in *International Conference on Smart Transportation and City Engineering (STCE 2024), Vol. 13575* (Bellingham, WA: SPIE), 541–547. doi: 10.1117/12.3062531

[B6] Gao R. Hu S. Yan L. Zhang L. Ruan H. Yu Y. . (2023). High-order deep infomax-guided deformable transformer network for efficient lane detection. Signal Image Video Process. 17, 3045–3052. doi: 10.1007/s11760-023-02525-y

[B7] Gu A. Dao T. (2023). Mamba: linear-time sequence modeling with selective state spaces. arXiv [preprint]. arXiv:2312.00752. doi” 10.4850/arXiv.2312.00752

[B8] He J. Ji Z. (2025). Decomposed spatio-temporal Mamba for long-term traffic prediction. AAAI Conf. Artif. Intell. 39, 11772–11780. doi: 10.1609/aaai.v39i11.33281

[B9] Ji S.-W. Lee J. Kim S.-W. Hong J. P. Baek S. Jung S. . (2022). “Xydeblur: divide and conquer for single image deblurring,” in 2022 IEEE/CVF Conference on Computer Vision and Pattern Recognition (CVPR) (New Orleans, LA: IEEE), 17400–17409. doi: 10.1109/CVPR52688.2022.01690

[B10] Kanopoulos N. Vasanthavada N. Baker R. L. (1988). Design of an image edge detection filter using the sobel operator. IEEE J. Solid-State Circuits 23, 358–367. doi: 10.1109/4.996

[B11] Kaushal R. K. Arind R, Giri, K. K. B. Sindhu M. Natrayan L. N. Ronald B. (2023). “Deep learning-based segmentation approach for automatic lane detection in autonomous vehicle,” in 2023 International Conference on Self Sustainable Artificial Intelligence Systems (ICSSAS) (Erode: IEEE), 268–273. doi: 10.1109/ICSSAS57918.2023.10331835

[B12] Kim Y. Denton C. Hoang L. Rush A. M. (2017). Structured attention networks. arXiv [preprint]. arXiv:1702.00887. doi: 10.48550/arXiv.1702.00887

[B13] Liang A. Jiang X. Sun Y. Lu C. (2024). Bi-mamba4ts: bidirectional Mamba for time series forecasting. arXiv [preprint]. arXiv:2404.15772. doi: 10.48550/arXiv.2404.15772

[B14] Lin L. Yan P. Xu X. Yang S. Zeng K. Li G. . (2021). Structured attention network for referring image segmentation. IEEE Trans. Multimed. 24, 1922–1932. doi: 10.1109/TMM.2021.3074008

[B15] Lin Y.-S. Chiang J.-C. (2024). “Hdrmamba: high dynamic range video reconstruction based on state space models,” in 2024 International Computer Symposium (ICS) (Piscataway, NJ: IEEE), 219–223.

[B16] Lin Z. Li M. Zheng Z. Cheng Y. Yuan C. (2020). Self-attention convlstm for spatiotemporal prediction. Proc. AAAI Conf. Artif. Intell. 34, 11531–11538. doi: 10.1609/aaai.v34i07.6819

[B17] Liu J. Gong M. Miao Q. Wang X. Li H. (2017). Structure learning for deep neural networks based on multiobjective optimization. IEEE Trans. Neural Netw. Learn. Syst. 29, 2450–2463. doi: 10.1109/TNNLS.2017.2695223 28489552

[B18] Liu L. Chen X. Zhu S. Tan P. (2021). “Condlanenet: a top-to-down lane detection framework based on conditional convolution,” in Proceedings of the IEEE/CVF International Conference on Computer Vision (Montreal, QC: IEEE), 3773–3782. doi: 10.1109/ICCV48922.2021.00375

[B19] Liu X. Zhang C. Zhang L. (2024). Vision Mamba: a comprehensive survey and taxonomy. arXiv [preprint]. arXiv:2405.04404. doi: 10.48550/arXiv.2405.04404 40982514

[B20] Liu Y. Huang T. Chen H. Zhang Y. Sun C. Li X. . (2021). “Lane structure fitting via row-wise grid classification,” in 2021 7th International Conference on Computer and Communications (ICCC) (Chengdu: IEEE), 599–604. doi: 10.1109/ICCC54389.2021.9674575

[B21] Liu Y. Wang J. Li Y. Li C. Zhang W. (2022). Lane-gan: a robust lane detection network for driver assistance system in high speed and complex road conditions. Micromachines 13:716. doi: 10.3390/mi13050716 35630183 PMC9142959

[B22] Luo X. Huang Y. Cui J. Zheng K. (2025). Deep learning-based lane detection for intelligent driving: a comprehensive survey of methods, datasets, challenges and outlooks. Neurocomputing 650:130795. doi: 10.1016/j.neucom.2025.130795

[B23] Mercy J. Lawanya R. Nandhini S. Saravanan M. (2022). “Effective image deblurring based on improved image edge information and blur kernel estimation,” in 2022 8th International Conference on Advanced Computing and Communication Systems (ICACCS), Vol. 1 (Coimbatore: IEEE), 855–859. doi: 10.1109/ICACCS54159.2022.9785087

[B24] Nazeri K. Ng E. Joseph T. Qureshi F. Z. Ebrahimi M. (2019). Edgeconnect: generative image inpainting with adversarial edge learning. arXiv [preprint]. arXiv:1901.00212. doi: 10.48550/arXiv.1901.00212

[B25] Patro B. N. Agneeswaran V. S. (2025). Mamba-360: survey of state space models as transformer alternative for long sequence modelling: methods, applications, and challenges. Eng. Appl. Artif. Intell., 159, 111279. doi: 10.1016/j.engappai.2025.111279

[B26] Pauly M. Keiser R. Gross M. (2003). Multi-scale feature extraction on point-sampled surfaces. Comput. Graph. Forum 22, 281–289. doi: 10.1111/1467-8659.00675

[B27] Qin Z. Wang H. Li X. (2020). “Ultra fast structure-aware deep lane detection,” in European Conference on Computer Vision (Cham: Springer), 276–291. doi: 10.1007/978-3-030-58586-0_17

[B28] Qin Z. Zhang P. Li X. (2022). Ultra fast deep lane detection with hybrid anchor driven ordinal classification. IEEE Trans. Pattern Anal. Mach. Intell. 46, 2555–2568. doi: 10.1109/TPAMI.2022.3182097 35696463

[B29] Rando S. Romani L. Migliarini M. Franco L. Gudovskiy D. Galasso F. . (2025). Serpent: selective resampling for expressive state space models. arXiv [preprint]. arXiv:2501.11729. doi: 10.48550/arXiv.2501.11729

[B30] Somvanshi A. Islam I. (2025). From s4 to Mamba: a comprehensive survey on structured state space models. arXiv [preprint]. arXiv:2503.18970. doi: 10.48550/arXiv.2503.18970

[B31] Song Y. Huang T. Fu X. Jiang Y. Xu J. Zhao J. . (2023). A novel lane line detection algorithm for driverless geographic information perception using mixed-attention mechanism resnet and row anchor classification. ISPRS Int. J. Geo-Inf. 12:132. doi: 10.3390/ijgi12030132

[B32] Su J. Chen C. Zhang K. Luo J. Wei X. Wei X. . (2021). Structure guided lane detection. arXiv [preprint]. arXiv:2105.05403. doi: 10.48550/arXiv.2105.05403

[B33] Su Y. Liu W. Yuan Z. Cheng M. Zhang Z. Shen X. . (2022). Dla-net: learning dual local attention features for semantic segmentation of large-scale building facade point clouds. Pattern Recognit. 123:108372. doi: 10.1016/j.patcog.2021.108372

[B34] Talib M. L. Rui X. Ghazali K. Zainudin N. M. Ramli S. (2013). “Comparison of edge detection technique for lane analysis by improved Hough transform,” in Communications in Computer and Information Science, eds. Zaman H. B. Robinson P. Olivier P. Shih T. K. Velastin S. (Cham: Springr), 176–183. doi: 10.1007/978-3-319-02958-0_17

[B35] Wang H. Hu C. Qian W. Wang Q. (2023a). Rt-deblur: real-time image deblurring for object detection. Vis. Comput. 40, 2873–2887. doi: 10.1007/s00371-023-02991-y

[B36] Wang Y. Jiang H. Jing Z. Chen G. Ma C. Qiu R. . (2023b). “Two-stage lane detection based on attention mechanism and post-processing of prediction,” in 2023 5th International Conference on Artificial Intelligence and Computer Applications (ICAICA) (IEEE), 274–279. doi: 10.1109/ICAICA58456.2023.10405438

[B37] Xi S. Liu Z. Wang Z. Zhang Q. Ding H. Kang C. C. . (2024). Autonomous driving roadway feature interpretation using integrated semantic analysis and domain adaptation. IEEE Access 12, 98254–98269. doi: 10.1109/ACCESS.2024.3429396

[B38] Xiao D. Zhuo L. Li J. Li J. (2021). Structure-prior deep neural network for lane detection. J. Vis. Commun. Image Represent. 81:103373. doi: 10.1016/j.jvcir.2021.103373

[B39] Xie Y. (2023). “Fast lane detection based on row anchor classification,” in Proceedings of the 2023 2nd International Conference on Networks, Communications and Information Technology (New York, NY: ACM). doi: 10.1145/3605801.3605823

[B40] Yang M. Wei Y. Pan L. Huang L. (2022). Exposure consistency for lane detection under varied light conditions. J. Electron. Imaging 31, 030502. doi: 10.1117/1.JEI.31.3.030502

[B41] Yurtsever E. Lambert J. Carballo A. Takeda K. (2020). A survey of autonomous driving: common practices and emerging technologies. IEEE Access 8, 58443–58469. doi: 10.1109/ACCESS.2020.2983149

[B42] Zakaria N. J. Shapiai M. I. Fauzi H. Elhawary H. M. Yahya W. J. Abdul Rahman M. A. . (2020). Gradient-based edge effects on lane marking detection using a deep learning-based approach. Arab. J. Sci. Eng. 45, 10989–11006. doi: 10.1007/s13369-020-04918-4

[B43] Zhang C. Wang F. Zhang X. Wang M. Wu X. Dang S. . (2025). MAMBA-CR: a state-space model for remote sensing image cloud removal. IEEE Trans. Geosci. Remote Sens. 63, 1–13. doi: 10.1109/TGRS.2024.35198102024.3519810

